# Nosocomial infections in an Iranian educational hospital: an evaluation study of the Iranian nosocomial infection surveillance system

**DOI:** 10.1186/s12879-021-06948-1

**Published:** 2021-12-15

**Authors:** Bagheri Pezhman, Rezaei Fatemeh, Roodgari Amir, Rokhsari Mahboobeh, Fararouei Mohammad

**Affiliations:** 1 Student Research Committee, Shiraz University of Medical Sciences, Shiraz, Iran; 2 Department of Social Medicine, Jahrom University of Medical Sciences, Jahrom, Iran; 3 Division of Infectious Disease, Department of Internal Medicine, Shiraz University of Medical Sciences, Shiraz, Iran; 4 Ali-Asghar Educational Hospital, Shiraz University of Medical Sciences, Shiraz, Iran; 5 HIV/AIDs Research Center, School of Health, Shiraz University of Medical Sciences, Razi street, Shiraz, Iran; 6 Non-communicable diseases Research Center, Fasa University of Medical Sciences, Fasa, Iran

**Keywords:** Cross-infection, Nosocomial infection, Infection rate

## Abstract

**Background:**

Nosocomial infection (NI) or cross-infection is a major health problem in hospitals worldwide.

**Aim:**

This study aimed to report the status of NIs and to evaluate the Iranian nosocomial infection surveillance system (INISS) in a teaching hospital in the south of Iran.

**Methods:**

This is a comparative historical study on the records of hospital admitted patients from 2018 to 2019. Data on patients who were diagnosed with NI was extracted from the INISS database. The database includes data on the incidence of different types of NIs in each hospital ward, the patient’s infection outcome, the agents involved, and the site of infection.

**Results:**

The results indicated that the rate of NI (cases of NI/ 100 admissions) in the hospital was %2.95. The highest rate of NIs was reported from ICUs. Of the infected patients, 45.61% were female, 98.95% had underlying diseases, and 30.88% died due to nosocomial infections. The median (IQR) of the duration of hospital stay among infected patients was 13 (7–18). The most common site of infection was VAE (ventilator-associated events) (39.40%) and the most common isolated agent, irrespective of the organ involved, was *Acinetobacter * (spp.) (22.75%).

**Conclusions:**

We reported ICU and *Acinetobacter * (spp.) as the most affected ward and most common agent involved in recorded NIs respectively. The rate of NI in the study hospital was exceptionally low when compared to its counterparts in a few other developed countries. The INISS needs to be further evaluated with regard to the completeness and representativeness of the surveillance system. Also, we need to evaluate the adherence to the INISS guidelines among staff and physicians in reporting the NIs.

## Introduction

Nosocomial infection (NI) or hospital-acquired infection is a common problem in hospitals worldwide [1]. Nosocomial infections cause major health problems including longer hospital stay and higher mortality among affected patients, especially in developing countries [2]. The rate of nosocomial infection is about 5% to 10% in industrialized countries, and about 20% to 25% in developing countries [3]. According to WHO, the highest rate of nosocomial infections is reported from the Eastern Mediterranean and Southeast Asia, and the lowest rate is reported from the Western Pacific, Ocean, and Europe [4]. It is also reported that about 7% of hospitalized patients in developed and 10% of them in developing countries acquire NIs [5]. According to several studies from the US, Europe, and Singapore, the incidence of NI varies from 3.2 to 11.9%, depending on the region [6–9]. Nosocomial infections increase hospital costs via longer hospital stay [3]. Also, patients with nosocomial infections can transmit the infection to other patients, increasing the risk of the spread of an invasive type of agent in the community [10]. It is estimated that NIs have imposed a cost of about $6127.65 in China in one year [11]. Finally, NIs are highly lethal depending on the characteristics of the infectious agents and the patients.

Despite the fact that NIs are quite common in medical centers, the diagnosis of NIs is not straightforward, as the organisms that are responsible for most nosocomial infections have dramatically changed over the past 30 years [12, 13]. That is why, despite the major progress in the prevention and treatment of infectious diseases, NIs continue to be the most important causes of mortality and adverse living conditions for millions of patients worldwide [14]. Arbitrary use of antibiotics in hospitals and communities not only causes microbial resistance that makes the treatment of NIs much harder and longer, but also causes many permanent health consequences in the affected patients [15]. As a result, monitoring and preventing NIs are key measures to improve the effectiveness and quality of healthcare. Accordingly, providing accurate and adequate information about NIs is essential in introducing effective prevention programs in hospitals [16]. This study aimed to represent the status of NIs and to evaluate INISS via reporting its main indexes and comparing the calculated NI rate in Ali-Asghar hospital (a referral teaching hospital in Shiraz, Iran) with few other hospitals worldwide.

## Methods

In this study, we analyzed the epidemiological status of nosocomial infections in a referral teaching hospital.

### Setting

Ali-Asghar is a teaching hospital under Shiraz University of Medical Sciences with 200 beds. The hospital is located in the south of Iran and has two intensive care units (ICUs) for internal diseases, one neurology ICU, and two general surgery wards as well as neurology, internal medicine, ophthalmology, gynecology, kidney, reconstructive and cosmetic surgery, ENT, poisoning, and several outpatient clinics. The hospital is a referral center for the above-mentioned specialties in the southern part of the country.

### The Iranian nosocomial surveillance system (INISS)

In each Iranian hospital, NIs are routinely registered with the INISS by a nosocomial infection control nurse who is well trained for monitoring the NI cases. The system was set up by the nosocomial infection care program under the direct supervision of the center for communicable disease control in the Iranian Ministry of Health and Medical Education. The system is used as a core approach for monitoring NIs in all Iranian hospitals. Currently, INISS has been integrated with the national health care program under the Iranian Ministry of Health. At the national level, the program has a technical committee managing the fields of scientific and executive issues, strengthening and expanding cross-sectoral coordination, programming, providing scientific support, and providing/ updating the guidelines [17]. The flowchart presents the workflow of the INISS according to the latest guidelines.

### Data collection process

**Fig. 1:**
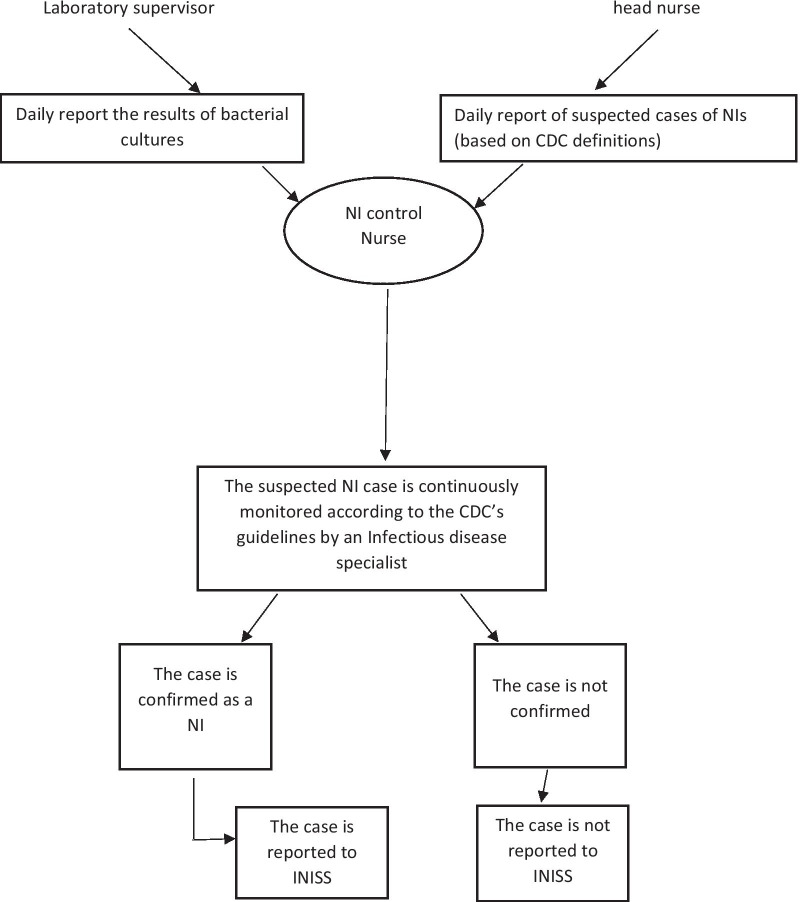
Algorithm for the registry of nosocomial infections with the Iranian nosocomial infection surveillance system [17]

Data was obtained from both the INISS database and the medical file of patients who were admitted to the hospital within the year preceding the starting time of the study (March 2019). The active lab based case finding is done based on the definitions provided by CDC under the National Healthcare Safety Network (NHSN) office and the national nosocomial infection registration program [18, 19]. According to the program (Fig. 1), the process of diagnosis and reporting NIs starts with the hospital’s infection control nurse collecting daily reports on suspicious cases of NI from the head nurses of all hospital wards based on criteria defined by CDC [20]. Also, the results of all positive microbial cultures or chest radiographies are reported by the supervisor of microbiological laboratory or a radiologist to the infection control nurse who later delivers the reports to the hospital’s well trained and experienced infectious disease specialist. The specialist investigates all reported cases and confirms the cases, providing the patients are on or after the 3rd hospital day and meet the predefined CDC’s criteria. If the suspicious cases of NI are confirmed by the specialist, the case is reported to the nosocomial infection nurse. Follow-up visits are paid by the infectious disease specialist to manage and monitor the condition in the NI patients from diagnosis to the date that the patient is discharged or dead. The data of the registered NI cases includes age, gender, date of hospitalization, the reason for the admission, date at which the first symptom of the NI was started the ICD code of the NI, the outcome of NI, type of organism found responsible for the infection, duration of hospital stay, the interval from hospital admission to the NI, hospital ward, the device involved, and the site of infection. To calculate the infection rate (incidence) of NIs, the number of nosocomial infections is divided by the number of admitted patients on a monthly basis [21–24]. Based on the ICD cods, total number of surgical site infections was divided by the number of registered surgeries conducted during the study period to calculate the surgical site infection rate. No data was available on the number of instrument used. As a result, the instrument specific infection was reported in percent of all instrument related infections.

In our study, the measured infection rate is compared with the corresponding index reported by other recently published studies (the past 10 years). The main criterion for including the articles was applying similar method of calculating the rate of NI among patients admitted to a general hospitals. Accordingly, databases of google scholar, web of science, Scopus, and PubMed were searched with the following search strategy: “infection rate OR incidence rate AND nosocomial infection OR hospital acquired infection” for finding relevant articles published from the year 2010 to 2020 (conducted before COVID-19 pandemic). Among the matched articles, several were excluded because they were conducted on specific types of patients, infections or conditions (e.g. coronavirus pandemic). Finally, 11 articles matched the inclusion criterion and used for the comparison. Median and percentage of numbers are used for descriptive analysis. To conduct the analysis, SPSS version 24 (IBM Corp, Armonk, NY) was used.

## Results

In total, 285 cases of nosocomial infections had been registered to INISS in the hospital from April 2018 to March 2019. The lowest and the highest age of patients who were diagnosed with nosocomial infection were 13 and 99 years respectively (the median (IQR) of patients’ age was 68.5 (62–71) years). The annual incidence rate of NI in the hospital was %2.95 and the highest NI rate was reported from the internal & neurology ICUs (37.02%) (Table 1). Table 2 presents the relative frequency NIs by sites, sex and duration of hospitalization. Accordingly, the most common sites reported to be involved with NI were ventilator-associated respiratory tract (39.30%), and UTI (Urinary tract infection) (33%). Of patients with NI, 30.88% died due to the infection. Of the reported NI cases, 45.61% were female and 98.95% had underlying diseases such as encephalitis, asthma, and diabetes. The median (IQR) of the duration of hospital stay among patients with NI was 13 (7–18) days (Table 2). Irrespective of the organ involved, the most common isolated microorganisms responsible for the NIs were *Acinetobacter* (spp.) (22.75%) and *Escherichia * (*E. coli*) (11.03%). However, the types of microorganisms involved in 32.41% of cases were not defined. Among all instrument related infections, endotracheal tubes (46.41%), and urinary catheters (32.49%) were the two most common devices involved. The rest of devices involved in the NIs were: other devices (13.8%), temporary central venous catheter (5.49%), peripheral venous catheter (1.69%), and arterial catheter (0.84%). More information regarding the frequency of infections by site and ward is given in Table 3. Also, as presented in Table 4, the nosocomial infection rates in the Iranian hospitals (including the current study), estimated based on CDC’s definitions, are among the lowest when compared with those reported from other countries (Table 4).

**Table 1:** Infection rate of nosocomial infection in different wards

Ward	No of nosocomial infections	Total number of hospitalized patients	NI Infection rate (%)
Internal & Neurology ICUs	204	551	37.02
Internal	29	1601	1.81
Neurology	21	1623	1.29
Surgery	25	3471^a^	0.72
CCU	3	947	0.32
Gynecology	3	1477	0.20
All	285	9670	2.95

*ICU* intensive care unit; *CCU* critical care unit

^a^Total number of recorded surgeries

**Table 2:** Frequency distribution of nosocomial infections by site, sex as well as duration of hospitalization

	VAE	PNEU	UTI	BSI	SSI	Other	Total
Total number of NIs (% of all sites)	112 (%39.3)	29 (%10.2)	94 (%33.0)	36 (%12.6)	12 (%4.2)	2 (%0.7)	285 (%100.0)
%Male	54 (%48.21)	20 (%68.97)	49 (%52.13)	20 (%55.56)	9 (%75.00)	1 (%50.00)	153 (%53.68)
Days of hospitalization Median (IQR)	19 (9–26)	10 (8–16)	17 (7–21)	12 (7–19)	7 (4–9)	14 (6–17)	13 (7–18)
Years of age Median (IQR)	45 (40–48)	73 (69.5–77)	67 (61–70)	70 (65–74)	69 (66–73)	59 (54–65)	68.5 (62–71)

*VAE* ventilator-associated events, *PNEU* non-ventilator-associated Pneumonia, *UTI* urinary tract infection, *BSI* bloodstream infection, *SSI* surgical site infection

**Table 3:** Frequency distribution of infections by site and ward

Ward	VAE (%)	PNEU (%)	UTI (%)	BSI (%)	SSI (%)	Others (%)	Total (%)	Admitted	Infection rate (%)
Internal ICUs	81 (62.8)	2 (1.6)	33 (25.6)	10 (7.8)	2 (1.6)	1 (0.8)	129 (100.0)	439	29.38
Neurology ICU	28 (37.3)	11 (14.7)	24 (32.0)	12 (16.0)	0 (0.0)	0 (0.0)	75 (100.0)	384	19.53
Surgery	0 (0.0)	1 (4.0)	17 (68.0)	0 (0.0)	7 (28.0)	0 (0.0)	25 (100.0)	3471	0.72
Women & Men ward	2 (6.3)	7 (21.9)	10 (31.3)	9 (28.1)	3 (9.4)	1 (3.1)	32 (100.0)	4624	0.69
CCU	0 (0.0)	0 (0.0)	2 (66.7)	1 (33.3)	0 (0.0)	0 (0.0)	3 (100.0)	1363	0.22
Neurology	1 (4.8)	8 (38.1)	8 (38.1)	4 (19)	0 (0.0)	0 (0.0)	21 (100.0)	2556	0.82

*ICU* intensive care unit; *CCU* critical care unit; *VAE* ventilator-associated events, *PNEU* non-ventilator-associated pneumonia, *UTI* urinary tract infection, *BSI* bloodstream infection, *SSI* surgical site infection

**Table 4:** Infection rate of nosocomial infection reported by the current study and some other recently published studies

Country	Publishing date	Most common site involved	Most common agent involved	Ward with the highest NI rate	Infection rate of NI (%)	Definition used
Iran*	2019	Respiratory tract	Acinetobacter	ICU^	2.95	CDC
China [25]	2018	Respiratory tract	*Pseudomonas aeruginosa*	Surgical ward	7.80	CDC
Greece [33]	2017	Lower respiratory tract infections	Klebsiella	Different hospital wards	5.2%	ECDC#
India [26]	2015	PNEU	Enterobacteriaceae	ICU	11.98	CDC
China [27]	2015	Respiratory tract	*Staphylococcus aureus*	ICU	7.57	CDC
India [31]	2015	pneumonia	*Klebsiella pneumonia*e	ICU	58.86	CDC
Iran [34]	2015	Urinary tract infection	*Escherichia coli*	Different hospital wards	< 2%	CDC
Gabon [29]	2014	surgical-site infections	*Staphylococcus aureus*	Surgical ward	1.60	CDC
Thailand [32]	2014	bacteremia	Acinetobacter	Different hospital wards	0.3	Study-based
Tunisia [28]	2014	PNEU	–	ICU	30.6	Study-based
India [30]	2013	Blood stream infections	*Streptococcus pneumonia*	ICU	17.6	CDC
Iran [2]	2013	Urinary tract infection	–	Surgical ward	4.14	CDC

*The current study

^^^Intensive Care Unit

^#^European Centre for Disease Prevention and Control

## Discussion

Nosocomial infections are the major causes of mortality in hospitalized patients. Effective control of NIs in health and medical centers requires sophisticated knowledge and understanding of the occurrence of the infections and their effective parameters [35]. Nosocomial infections not only cause morbidity and mortality but also impose a high cost to the health system and the affected patients [36–38]. The results of this study indicated that the infection rate of NIs was %2.95, a rate much lower than the estimates provided by studies from other countries. Based on the results of a meta-analysis in 2014, based on the INISS independent sources of data, the overall estimated incidence of NI in Iran was about 30.4% [39], a completely different figure compared to what was observed in the current study. Similarly, another systematic review on the studies in Tehran (the capital) in 2017 [40], suggested that the incidence rate of NI was about 21.85%, again a figure much higher than the results of the current study. Despite the fact that a set of public and private and non-Teaching hospitals in Iran were included in the above studies, the figures suggest that the rate of NI in our study hospital is meaningfully lower. The disparity can suggest a problem in the validity of the reported figures from INISS. However, despite the observed discrepancy, according to the results from the meta-analysis [39], the most common sites of NI were the respiratory tract (39.4%), urinary tract (23.8%), and bacteremia (21.9%), a pattern similar to what was observed in the current study. However, based on the results of the other meta-analysis [40], the most common causes of NIs in Tehran’s non-teaching hospitals were *Klebsiella pneumonia* (31.4%), *Escherichia coli* (30.9%), *Pseudomonas aeruginosa* (26.7%), and Staphylococcus (23.6%) [40], a pattern significantly different from what was observed in the current study. In the present study, Acinetobacter and *Escherichia coli* were the most common microorganisms isolated from the patients. With regard to the NIs reported from the hospital wards, the results of the review [40] suggested that the highest rates of incidence of NIs were reported from ICU, surgical (Gynecology, general and neonatal), and burn units respectively. In the present study, the highest rates of infection were reported in the internal ICU and neurology ICU. The results of other Iranian studies on the epidemiological status of NIs in terms of the wards and the involved infectious agents are relatively similar to the results of the present study [36, 41–43]. Accordingly, the results of the current study and other studies from different parts of the country are different in terms of the agent, and site of NIs. Differences in the environment, building, staff, and patient’s underlying diseases and behaviors may, to some extent, explain the observed differences.

According to the results of the current study, the intensive care unit has the highest rate of NI compared to the other units of the hospital. This finding is consistent with few other studies from different parts of the world [44–47]. The high rate of NI and its related deaths among ICU admitted patients are closely related to the clinical status of patients who undergo invasive medical and resuscitate procedures. With regard to the mode of transmission, according to an estimate, 25% of all hospital infections in ICUs are transmitted via blood and air [48] a pattern relatively different from what was reported by the current study.

In the present study, the most common instruments contributing to NIs were ventilators, endotracheal tubes, and urinary catheters. In a study by Rahmanian, the most common factors contributing to NIs were catheters and surgical wounds [49]. Continuous hygiene monitoring by the infection control nurses may enhance the effect of the cleaning process and reduce the risk of infection transmission [50]. In a study in Tehran [40], the most common agents involved in NIs that were transmitted by the use of different types of catheters were resistant to the commonly available antibiotics. Disinfection and sterilization of medical supplies and equipment are always a big challenge in hospitals and clinics even though they are known as the main vehicles involved in NIs. Putting beside the possibility of lower NIs transmission in Iranian hospitals, the lower reported NI rates of the two Iranian hospitals (including the present study) when compared with the other selected studies may suggest a problem in the completeness of the system. It is also worth noticing that in Iran, a significant number of surgeons used antibiotics as a nosocomial infection preventive measure, an intervention that may significantly reduce the number of NIs.

## Conclusion

The overall findings of the present study indicated that the incidence, etiology, and causes of nosocomial infections in the studied educational hospital are significantly different when compared to those reported from other hospitals. Using data from INISS, the rate of NI in the study hospital was exceptionally lower than its counterparts in Iran and some other countries, which used sources of data other than INISS. Also, we noticed a significant discrepancy between the agents involved in NIs when compared to hospitals in other countries. The INISS needs to be further evaluated regarding completeness and representativeness. Also, adherence to the guidelines among staff and physicians in reporting the NIs as well as the effect of structural and instrumental factors on the diagnosis of NIs may explain the observed pattern. It seems that interventions including supervising staffs regarding their adherence to the defined guidelines and more physicians and nurses training with regard to the diagnosis and reporting nosocomial infections are suggested [51].

### Limitations

No follow-up was made to the patients after discharge. This causes a possibly significant underestimation of the incidence of NIs. We could not calculate the device associated infection rate due to not having the data on devices usage by admitted patients.

## Data Availability

The datasets used and/or analyzed during the current study are available from the corresponding author on reasonable request.
